# Persistent cortical and white matter inflammation after therapeutic hypothermia for ischemia in near-term fetal sheep

**DOI:** 10.1186/s12974-022-02499-7

**Published:** 2022-06-11

**Authors:** Kelly Q. Zhou, Laura Bennet, Guido Wassink, Alice McDouall, Maurice A. Curtis, Blake Highet, Taylor J. Stevenson, Alistair J. Gunn, Joanne O. Davidson

**Affiliations:** 1 Department of Physiology, School of Medical Sciences, The University of Auckland, 85 Park Road, Grafton, Auckland, 1023 New Zealand; 2 Department of Anatomy and Medical Imaging, School of Medical Sciences, The University of Auckland, Auckland, New Zealand; 3 Department of Pharmacology, School of Medical Sciences, The University of Auckland, Auckland, New Zealand

**Keywords:** Hypoxia-ischemia, Therapeutic hypothermia, Neuroinflammation, Microglial phenotype, Gitter cells, Electroencephalogram

## Abstract

**Background:**

Therapeutic hypothermia significantly improves outcomes after moderate–severe hypoxic-ischemic encephalopathy (HIE), but it is partially effective. Although hypothermia is consistently associated with reduced microgliosis, it is still unclear whether it normalizes microglial morphology and phenotype.

**Methods:**

Near-term fetal sheep (*n* = 24) were randomized to sham control, ischemia-normothermia, or ischemia-hypothermia. Brain sections were immunohistochemically labeled to assess neurons, microglia and their interactions with neurons, astrocytes, myelination, and gitter cells (microglia with cytoplasmic lipid granules) 7 days after cerebral ischemia. Lesions were defined as areas with complete loss of cells. RNAscope^®^ was used to assess microglial phenotype markers *CD86* and *CD206*.

**Results:**

Ischemia-normothermia was associated with severe loss of neurons and myelin (*p* < 0.05), with extensive lesions, astrogliosis and microgliosis with a high proportion of gitter cells (*p* < 0.05). Microglial wrapping of neurons was present in both the ischemia groups. Hypothermia improved neuronal survival, suppressed lesions, gitter cells and gliosis (*p* < 0.05), and attenuated the reduction of myelin area fraction. The “M1” marker *CD86* and “M2” marker *CD206* were upregulated after ischemia. Hypothermia partially suppressed *CD86* in the cortex only (*p* < 0.05), but did not affect *CD206.*

**Conclusions:**

Hypothermia prevented lesions after cerebral ischemia, but only partially suppressed microglial wrapping and M1 marker expression. These data support the hypothesis that persistent upregulation of injurious microglial activity may contribute to partial neuroprotection after hypothermia, and that immunomodulation after rewarming may be an important therapeutic target.

## Background

There is now compelling evidence that therapeutic hypothermia for hypoxic-ischemic encephalopathy (HIE) significantly reduces grey and white matter lesions in the brain [1, 2] and improves survival without disability [3–5]. However, in a recent trial, 29% of infants with HIE died or developed moderate-to-severe disability despite hypothermia [6]. In order to develop effective add-on treatment strategies to further reduce this burden of death and disability, it is important to better understand the mechanistic limitations of therapeutic hypothermia that may contribute to its partial or lack of protective effects in some cases. We have previously shown that after severe global cerebral ischemia in near-term fetal sheep, although therapeutic hypothermia started at 3 h after the end of ischemia was associated with near-complete protection of neurons, there was a persisting deficit in electroencephalogram (EEG) power compared with sham control animals [7].

In this setting, it is intriguing to note that there is evidence from animal studies for persistent neuroinflammation despite therapeutic hypothermia [8–10]. The impact of the inflammatory response after hypoxia-ischemia (HI) is rather complex. Microglia are rapidly upregulated after HI, and contribute to secretion of cytokines and clearance of cellular debris [9, 11–14]. The net effect of microglial induction seems to be beneficial as shown by exacerbation of brain injury when microglia were depleted in postnatal day 10 (P10) mice after common carotid artery ligation plus hypoxia [15]. Nevertheless, in studies in P9 mice, HI was associated with significant upregulation of both pro and anti-inflammatory genes from 24 h [16, 17]. Supporting the hypothesis that activation of these pro-inflammatory genes is deleterious, in P10 mice, administration of the anti-inflammatory factor colony-stimulating factor 1 after HI was neuroprotective [18]. Potentially, these changes may be a target for therapeutic hypothermia; in P9 mice HI was associated with upregulation of the “pro-inflammatory” marker *CD86* and the “anti-inflammatory” marker *CD206* at day 1, while immediate hypothermia for just 4 h after HI was associated with suppression of the pro-inflammatory response with reduced infiltration of peripheral macrophages [17].

Further, astrocytes also are involved in immune signaling after HI [19–21]. For example, preventing astrocyte activation by knocking out transient receptor potential vanilloid 1 reduced inflammation and improved brain injury after common carotid artery ligation and hypoxia in mice [22]. Conversely, however, astrocytes in vitro promoted release of neurotrophic factors that promoted neuronal survival after oxygen glucose deprivation [23]. Both cortical and white matter astrocytes underwent significant morphological changes in neonatal piglets after exposure to 30 min of 4% oxygen [21].

To the best of our knowledge, there have been no studies of the impact of therapeutic hypothermia on expression of the microglial M1 “pro-inflammatory” and M2 “anti-inflammatory or reparative” phenotypes [24] in a large translational animal model of HI and therapeutic hypothermia. The key aim of the current study was to determine whether a clinical protocol of delayed, but prolonged hypothermia can normalize microglial morphology and phenotype after 7 days recovery from global ischemia in near-term fetal sheep, at an age when neural maturation is broadly similar to the term human infant [25]. A secondary aim was to relate changes in microglial and astrocyte morphology to the pattern of cortical and white matter injury.

## Methods

### Surgery

All the procedures were approved by the Animal Ethics Committee of The University of Auckland, under the New Zealand Animal Welfare Act and the Code of Ethical conduct for animals in research established by the Ministry of Primary Industries, Government of New Zealand. This study has been reported in compliance with the ARRIVE guidelines.

24 time-mated Romney/Suffolk fetal sheep were instrumented at 125 ± 1 days gestation (term = 145 days) under sterile conditions [7, 26]. Food but not water was withdrawn from the ewes 18 h prior to surgery. Ewes were administered long-acting oxytetracycline i.m. (20 mg/kg, Phoenix Pharm, Auckland, New Zealand) at 30 min before the start of surgery. Propofol i.v. (5 mg/kg, AstraZeneca Limited, Auckland, New Zealand) was used to induce anesthesia and maintained using 2–3% isoflurane in O_2_ during surgery. The depth of anesthesia, maternal respiration and heart rate were monitored by trained staff. Ewes were on a continuous infusion of isotonic saline at approximately 250 mL/h for the maintenance of fluid balance.

Fetuses were partially exteriorized after a maternal abdominal and uterine incision. The fetus was instrumented for continuous data recording of physiological parameters. Equipment relevant to this study included inflatable carotid artery occluders placed around both carotid arteries, after ligating the vertebral–occipital anastomoses and two pairs of 7 stranded stainless steel wire electroencephalograph electrodes placed on the dura over the cortex of the parasagittal gyrus (10 mm and 20 mm anterior to bregma and 10 mm lateral) and secured with cyanoacrylate glue. The reference electrode was sewn over the occiput. A thermistor was placed on the dura over the cortex of the parasagittal gyrus to measure extradural temperature and another thermistor was inserted into the esophagus to measure body temperature. A cooling cap made out of silicon tubing (3 × 6 mm, Degania Silicone, Israel) was secured on the fetal head.

Fetuses were returned to the uterus and the uterine incision was closed. Antibiotics (80 mg Gentamicin, Pharmacia and Upjohn, Rydalmere, New South Wales, Australia) were administered into the amniotic sac. 10 mL 0.5% bupivacaine plus adrenaline (AstraZeneca Ltd., Auckland, New Zealand) was injected in the maternal laparotomy skin incision. All fetal catheters and leads were exteriorized through the maternal flank. The maternal long saphenous vein was catheterized for post-operative maternal care and euthanasia.

### Post-operative care

Sheep were housed together in individual metabolic cages with ad libitum access to food and water. The rooms were temperature controlled (16 ± 1 °C, humidity 50 ± 10%), with a 12 h light/dark cycle. Antibiotics were administered i.v. daily to the ewe (600 mg benzylpenicillin sodium for 4 days, Novartis Ltd, Auckland, New Zealand and 80 mg gentamicin for 2 days). A continuous infusion of heparinized saline was used to keep fetal catheters patent (20 U/mL at 0.15 mL/h) and the maternal catheter was maintained by daily flushing.

### Data recordings

Data recordings began 24 h before the start of experiments and continued for the duration of the experiments for 7 days. Data were recorded and saved continuously for off-line analysis using custom-made acquisition programs LabView for Windows (National Instruments, Austin, TX, USA).

### Experimental protocols

All fetuses had normal biochemical variables for their gestational ages before the start of the experiment. Fetuses were randomized to sham control (*n* = 8), ischemia-normothermia (*n* = 8) or ischemia-hypothermia (*n* = 8). At 129 ± 1 day gestation, ischemia was induced by reversible inflation of the carotid artery occluders for 30 min with sterile saline. In the sham control group, carotid artery occluders were not inflated. Fetal blood samples were taken before the occlusion and at 2, 4 and 6 h after occlusion, then daily for the remainder of the experiment for pre-ductal pH and blood gas (ABL800 Flex Analyzer, Radiometer, Auckland, New Zealand), and glucose and lactate measurements (YSI model 2300, Yellow Springs, Ohio, USA).

Cooling was initiated in the ischemia-hypothermia group at 3 h after the end of ischemia and continued until 72 h. Cooling was achieved by attaching the cooling coil placed on the fetal head to a pump circulating cold water through the cooling coil. The targeted extradural temperature was between 31 and 33 °C in the first 1–2 h. After the cooling period, the cooling machine was turned off and fetuses were allowed to rewarm spontaneously, over < 1 h. In the sham control and ischemia-normothermia groups, the water was not circulated through the cooling coil.

Ewes and fetuses were killed at 7 days after cerebral ischemia with an overdose of sodium pentobarbitone (9 g i.v. to the ewe; Pentobarb 300; Chemstock, Christchurch, New Zealand). Fetal brains were perfusion fixed with 10% phosphate-buffered formalin and embedded in paraffin for immunohistochemistry and RNAscope^®^.

### Immunohistochemistry

#### Diaminobenzidine tetrachloride (DAB) immunohistochemistry

Coronal sections (10 µm thick) were cut at the mid-striatal level, using a microtome (Leica Jung RM2035, Wetzlar, Germany). Serial sections were obtained and one section was used for each marker or combination of markers. Sections were dried in an oven for 4 h at 60 °C, followed by dewaxing in xylene and rehydration in decreasing concentrations of ethanol and then washed in 0.1 M phosphate-buffered saline (PBS). Antigen retrieval was performed using a pressure cooker (2100 Antigen Retriever, Aptum Biologics Ltd., Southampton, England) in 0.1 M citrate buffer. DAB immunohistochemistry sections, were incubated in 1% H_2_O_2_ in methanol for 30 min to block endogenous peroxidase activity. Blocking was performed in 3% normal goat serum (NGS) for 1 h at room temperature. Sections were labeled with 1:200 rabbit anti-NeuN (Cat# MAB377 Millipore, Burlington, Massachusetts, USA), 1:200 mouse anti-MBP (Cat# MAB381, Millipore), 1:200 rabbit anti-Iba1 (Cat# ab178846, Abcam, Cambridge, England), or rabbit anti-GFAP (Cat# ab68428, Abcam) and incubated overnight at 4 °C. Sections were incubated in 1:200 biotin-conjugated goat anti-mouse (Cat# BA-9200, Vector Laboratories, Burlingame, CA, USA), or 1:200 biotin-conjugated goat anti-rabbit (Cat# BA-1000, Vector Laboratories) for 3 h, then incubated in 1:200 ExtrAvidin (Cat# E2886, Sigma-Aldrich Pty. Ltd, St Louis, USA) for 2 h at room temperature. Sections were reacted with 3,3ʹ-diaminobenzidine tetrahydrochloride (DAB, Sigma-Aldrich). The reaction was stopped by washing in PBS. Sections were dehydrated in alcohol and xylene and mounted in DPX mounting medium (Sigma).

### Fluorescence immunohistochemistry

The dewaxing, rehydration and antigen retrieval procedure was carried out as above. Sections were labeled with the appropriate combination of 1:200 mouse anti-MBP (Cat# MAB381, Millipore), 1:200 rabbit anti-Iba1 (Cat# ab178846, Abcam, Cambridge, England), or rabbit anti-GFAP (Cat# ab68428, Abcam), incubated overnight at 4 °C. The appropriate secondary antibodies were added; Alexa Fluor 488 goat anti-rabbit (Cat# A11008, Invitrogen, Carlsbad, California, USA), Alexa Fluor 568 goat anti-mouse (Cat# A11004, Invitrogen) and/or Alexa Fluor 647 goat anti-rabbit (Cat# A32733 Invitrogen) (1:200) incubated for 3 h at room temperature. Hoechst (Sigma) was applied to sections for 10 min, then mounted with Vectashield (Vector Laboratories).

#### Terminal deoxynucleotidyl transferase dUTP nick end labeling (TUNEL) assay with immunohistochemistry

After dewaxing and rehydrating sections and antigen retrieval, TUNEL solution (1 part enzyme solution to 10 parts label solution) (In Situ Cell Death Detection Kit, Fluorescein conjugated, Roche, Basel, Switzerland) was added to sections. Sections were covered with parafilm and incubated at 37 °C for 2 h. Sections were washed with PBS and blocked with NGS, then fluorescence immunohistochemistry was performed as above.

#### RNAscope^®^ with immunohistochemistry

The formalin-fixed paraffin-embedded tissue sections were dried in an oven for 4 h at 60 °C and dewaxed in xylene (2 × 15 min). RNA in situ hybridization was performed using the RNAscope^®^ 2.5 HD Assay—RED (Advanced Cell Diagnostics, Newark, CA, United States), as per manufacturer instructions. In brief, sections were placed in 100% ethanol (2 × 5 min) and air dried. Hydrogen peroxide solution (Advanced Cell Diagnostics) was added to the sections and incubated for 10 min at room temperature. Sections were washed two times in MilliQ. Antigen retrieval was performed by boiling sections in 1× Target Antigen Retrieval Reagents (Advanced Cell Diagnostics) for 15 min at 98–102 °C and washed in MilliQ. Sections were transferred into 100% ethanol for 3 min and incubated in the oven at 60 °C for an hour. Protease Plus solution (Advanced Cell Diagnostics) was added to the sections, followed with incubation for 30 min in the HybEZ II Hybridization System Oven (Advanced Cell Diagnostics) at 40 °C and washing MilliQ. Custom-made ovine specific probes to detect cluster of differentiation *(CD)86* and *CD206* mRNA (Advanced Cell Diagnostics) were added and incubated at 40° for 2 h. Amp 1 (30 min at 40 °C), Amp 2 (15 min at 40 °C), Amp 3 (30 min at 40 °C), Amp 4 (15 min at 40 °C), Amp 5 (30 min at room temperature) and Amp 6 (15 min at room temperature) were applied (Advanced Cell Diagnostics). Sections were washed twice in 1× Wash buffer (Advanced Cell Diagnostics) between each step. The RNAscope^®^ signal was detected by adding one part Red B solution to 60 parts Red B solution (Advanced Cell Diagnostics) and incubating for 15 min at room temperature. Sections were washed in MilliQ. This was followed by procedures as described for fluorescence immunohistochemistry, starting with blocking with the appropriate serum. All tissue used in this study stained positive for the low copy number, rigorous positive control probe for ovine *POLR2A* and showed negative staining with the negative control probe for *Bacillus subtilis DapB.*

### Imaging

#### Brightfield Vslide imaging

DAB-labeled sections for NeuN, MBP, Iba1 and GFAP were imaged on the Zeiss Axio Imager Z2 (MetaSystems Metafer5 VSlide software, Boston, MA, United States) using the 10× objective (0.45 numerical aperture), with the green transmitted light filter (GTL). The whole coronal section was imaged and stitched using the Metacyte software (Metasystems).

#### Fluorescence and confocal imaging

Fluorescence images of Hoechst, Iba1 and MBP triple labeling were acquired on the Zeiss Axio Imager M2 using the 40× and 63× objectives (Carl Zeiss AG, Oberkochen, Germany), with the ZEISS Apotome 2 (Carl Zeiss), in a z-stack. Confocal images of Hoechst, NeuN, Iba1 and TUNEL quadruple labeling were acquired using the FV1000 confocal microscope (Olympus, Japan) with a 40× magnification oil immersion lens (1.00 NA), in a Z-stack.

#### Fluorescence combined with brightfield Vslide imaging

RNAscope and immunohistochemically labeled sections were imaged on the Zeiss Axio Imager Z2 (MetaSystems Metafer5 VSlide software) using the 10× objective (0.45 numerical aperture). As shown by Highet et al. the RNAscope Fast Red chromogen is visible under both transmitted light (GLT) and the TexasRed (TxRed) filter [27]. Therefore, to minimize the effect of autofluorescence and tissue artifacts, the RNAscope^®^ puncta were imaged under GTL and TxRed filters. The Hoechst and Iba1 labeling (Cy7) were imaged with their respective filters. The whole first parasagittal gyrus was imaged and stitched using the Metacyte software (Metasystems).

### Image analysis

#### Analysis of DAB images

**Fig. 1:**
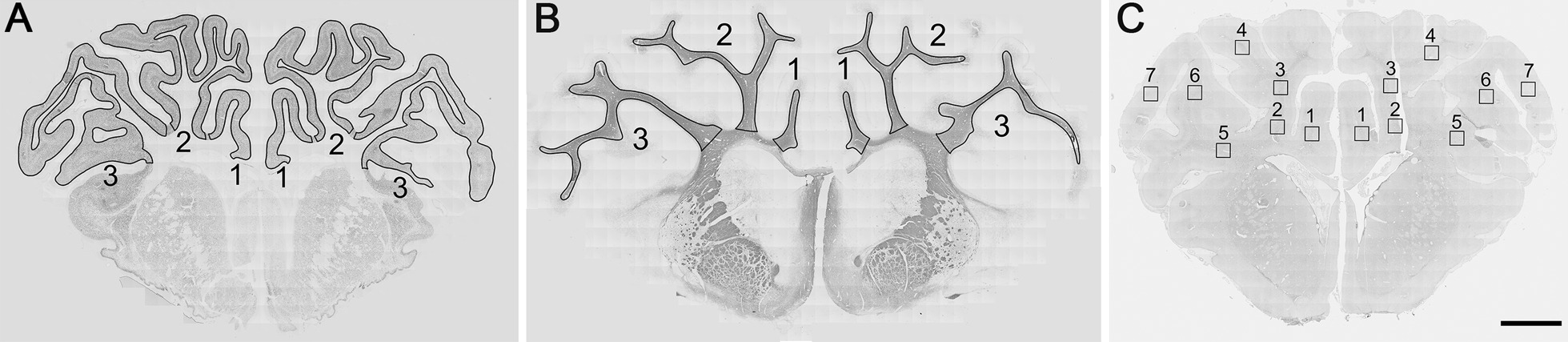
Diagram of tracing and imaging areas. **A** The NeuN areas traced for area fraction and area measurements for the cortex of the (1) sagittal, (2) first parasagittal and (3) second parasagittal gyri. **B** The MBP areas traced for area fraction and area measurements for the intragyral white matter of the (1) sagittal, (2) first parasagittal and (3) the second parasagittal gyri. **C** The locations of where images were extracted for counting for (1) the intragyral white matter of the sagittal gyrus (2) the base and (3) the middle and (4) the top of the intragyral white matter of the first parasagittal gyrus and (5) the base, (6) the middle and (7) the top of the intragyral white matter of the second parasagittal gyrus. Scale bar = 5 mm

Area fraction and area measurements of cortical NeuN labeling was performed by tracing around the sagittal and the first and second parasagittal gyri as shown (Fig. 1A). MBP area fraction and area fraction measurements of MBP labeling was performed by tracing around the white matter of the sagittal gyrus and the intragyral white matter of the first and second parasagittal gyri (Fig. 1B). GFAP cortical and white matter areas were traced in a similar manner. Area fraction was measured after using the default function of autothresholding on FIJI. For cell counting, 670 × 670 µm images were extracted using the VSviewer software (MetaSystems) from predetermined regions of interest in the sagittal and the first and second parasagittal gyri (Fig. 1C). Cell counts are the averages of two regions of interest for the sagittal gyrus, and the averages of the six regions of interest for the first and second parasagittal gyri.

Cortical and white matter lesions were defined as macroscopically evident irregular architecture or cellular loss and not spaces due to the presence of blood vessel or any artifacts in tissue processing or during the immunolabeling process. Areas outside of these lesions were defined as intact tissue. Extensive diffuse MBP loss in the white matter were defined as sparse MBP labeling present throughout the intragyral white matter of the parasagittal gyri. Localized diffuse MBP loss was defined as sparse MBP labeling in some regions only of the intragyral white matter of the parasagittal gyri. For cell counting in lesion, extensive and localized diffuse MBP loss areas, 340 µm × 340 µm images were extracted using the VSviewer software (MetaSystems). For cell counting in the intact areas, images of the same size were extracted from the base of the intragyral white matter of the first parasagittal, for consistency across different animals.

Cell number was quantified using FIJI by manual counting of positively labeled cells. For Iba1 and GFAP labeling, all positive cells with visible cell bodies were included. Iba1 and GFAP-positive cell bodies were counted and expressed as number/field of view.

#### Analysis for fluorescence images

Maximal projections of Hoechst, Iba1 and MBP triple-labeled z-stack images from the base, the middle and the top of the cortex and intragyral white matter of the first parasagittal gyrus were used to better visualize microglial morphology using FIJI. All Iba1-positive cells containing nuclei were counted, and a subset of Iba1-positive gitter cells (identified as containing two or more vacuoles) were also counted. For images of NeuN labeled with Hoechst and either Iba1 or GFAP, area fractions were measured after using the default function for Hoechst, Triangle for NeuN and GFAP and Moments for Iba1 for autothresholding on FIJI. For NeuN labeling, healthy NeuN-positive cells were defined as having typical nuclei that were not condensed or fragmented.

#### RNAscope^®^ analysis

The RNAscope^®^ analysis was adapted from [27]. Images were extracted using the VSviewer software (MetaSystems) from the base, the middle and the top of the cortex and the intragyral white matter of the first parasagittal gyrus for analysis in an uncompressed 8-bit TIFFs format using FIJI. The averages of the three regions of interest were calculated for both the cortex and the intragyral white matter of the first parasagittal gyrus. A Difference of Gaussian (DoG) based background subtraction (Gaus01 *Ω* = 0.6, Gaus02 *Ω* = 5.0) was used to smooth single pixel variation and set the upper limit for puncta identification. The image was auto-thresholded using the Triangle method and converted to a mask. Puncta were then cleared and dilated by 5 pixels (1.8 µm^2^) to compensate for alignment errors between GTL and TxRed filters. The TxRed image was processed as above but clearing and dilating puncta were not performed. Instead, watershed separation was performed on the puncta masks and single pixels were filtered. Overlapping puncta between GTL and TxRed images were found by combining the two processed images using the ‘AND’ function. Only puncta with a minimum size of 4 pixels were included. The final output presented was the area of puncta staining as a percentage of the total area of the image (area fraction).

### Statistical analysis

For the M1 marker, given a population SD of 1.8, a sample size of 4 or more per group was required to provide 98% power to detect a twofold difference. For the M2 marker, given a population SD of 0.48, a sample size of at least 4 per group was required to provide 95% power to detect a 20% difference. The Shapiro–Wilk test was used to assess normality of data distribution. When multiple areas were assessed, a repeated measures analysis of variance (ANOVA) was performed with the different areas treated as repeated measures, followed by a Fisher’s least significant difference (LSD) post hoc test when statistical significance was found (IBM SPSS Statistics 24). The Fisher’s exact test was used to assess differences in the number of animals with lesions and white matter injury. Non-parametric data were assessed with the Kruskal–Wallis test. Linear regression was used to analyze histological and physiological correlations within subjects (GraphPad Prism 7). For all other remaining data, one-way ANOVA was used, followed by a LSD post hoc test when statistical significance was found (IBM SPSS Statistics 24). Statistical significance was accepted when *p* < 0.05.

## Results

### Demographic data

There were no significant differences in sex, the proportions of singleton to twins and fetal body weight at post-mortem between the ischemia groups, but a higher proportion of twins in the sham control groups (Table 1). There was a reduction in brain weight in the ischemia-normothermia group compared with sham control (*p* = 0.001), which was attenuated to sham control values in the ischemia-hypothermia group (*p* = 0.125).

**Table 1:** Demographic data for sham control, ischemia-normothermia and ischemia-hypothermia groups

Group	Sex	Body weight (g)	Brain weight (g)	Singleton/twin
Sham control	4 M, 4 F	4936 ± 311	48.1 ± 2.0	1 singleton, 7 twins^^#^
Ischemia-normothermia	3 M, 4 F, 1 U	4575 ± 311	40.1 ± 1.5*	7 singleton, 1 twin
Ischemia-hypothermia	4 M, 4 F	4720 ± 145	44.9 ± 0.9^	7 singleton, 1 twin

M = male, F = female, U = unknown. *(*p* < 0.05) vs. sham control, ^^^(*p* < 0.05) vs. ischemia-normothermia, ^#^(*p* < 0.05) vs. ischemia-hypothermia, one-way ANOVA and LSD post hoc test for weights, Kruskal–Wallis one-way ANOVA for the distribution of sex and singleton/twins

### EEG recovery

Changes in EEG power data have been previously published [7]. The ischemia-normothermia group had profoundly suppressed EEG power at day 7 of recovery after ischemia (Table 2). The ischemia-hypothermia group showed improved EEG recovery compared with ischemia-normothermia (*p* = 0.001).

**Table 2:** Final delta EEG power at day 7 after ischemia, compared with baseline

	Ischemia-normothermia	Ischemia-hypothermia
Delta EEG power at day 7 (dB)	− 11.8 ± 1.5	− 1.4 ± 1.2^^^

^^^(*p* < 0.05) vs. ischemia-normothermia, independent samples *T*-test

### Cortical and white matter area and lesions

**Fig. 2:**
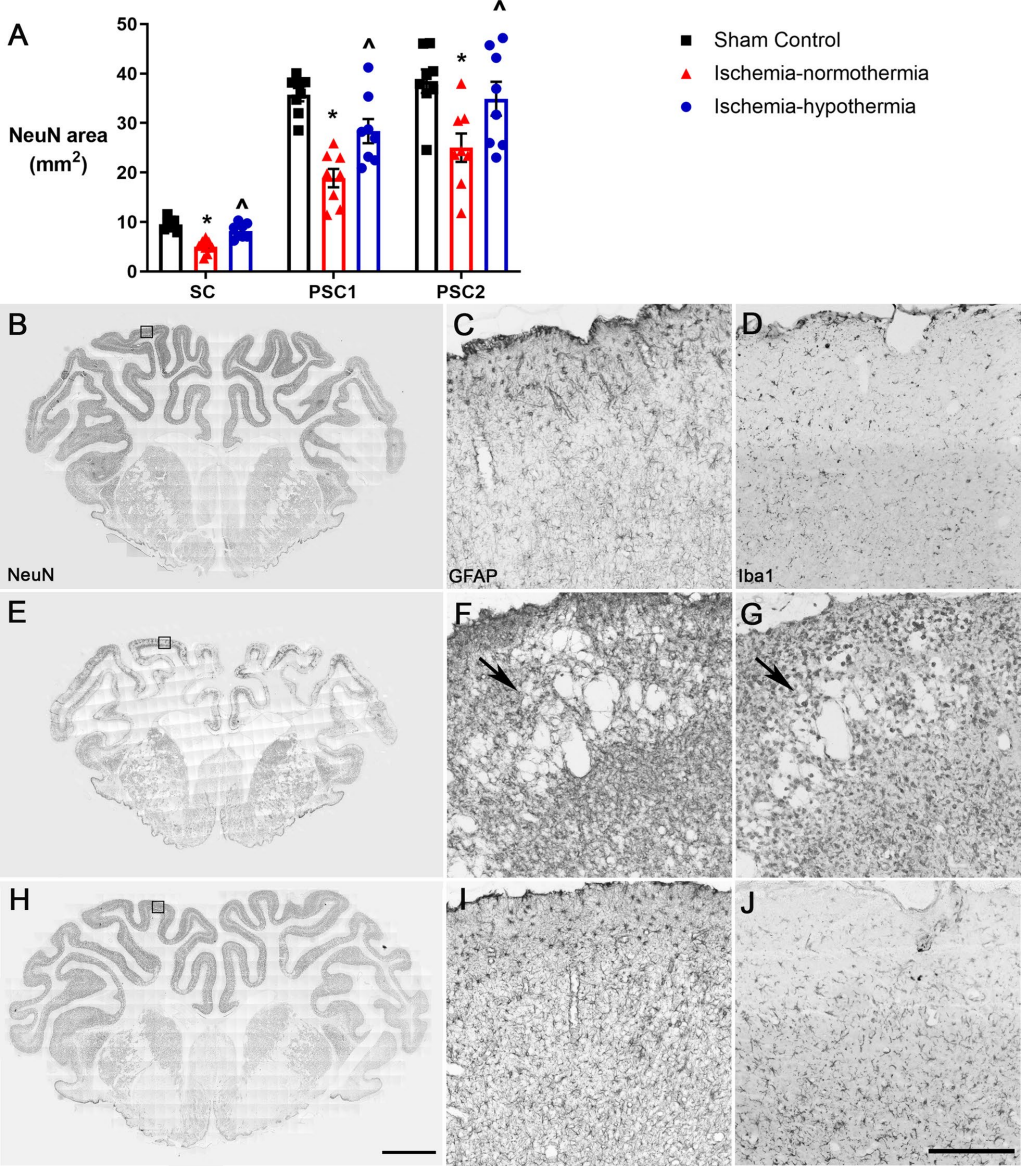
Cortical neuronal area and cortical lesions. **A** Graph showing NeuN area measurements for the cortex of the sagittal (SC), first parasagittal (PSC1) and second parasagittal gyri (PSC2). Representative NeuN whole section images for **B** sham control (*n* = 8), **E** ischemia-normothermia (*n* = 8) and **H** ischemia-hypothermia (*n* = 8). Representative GFAP images in the cortex taken from area denoted by the box for **C** sham control showing no lesions, **F** ischemia-normothermia showing lesions and **I** ischemia-hypothermia showing no lesions. Representative Iba1 images in the cortex for **D** sham control showing no lesions, **G** ischemia-normothermia showing lesions and **J** ischemia-hypothermia showing no lesions. Arrows indicate lesions. *(*p* < 0.05) vs. sham control, ^^^(*p* < 0.05) vs. ischemia-normothermia, repeated measures ANOVA and LSD post hoc test. Scale bar for **F** = 5 mm (NeuN), scale bar for **J** = 200 µm (GFAP and Iba1)

Area of NeuN labeling in the cortex of the sagittal and the first and second parasagittal gyri was significantly reduced in the ischemia-normothermia group compared with sham control (Fig. 2; *p* = 0.001). The reduction in cortical area was significantly attenuated in the ischemia-hypothermia group to near sham control values (*p* = 0.084). Cortical lesions were present in 7/8 of the ischemia-normothermia animals (*p* = 0.001), whereas none of the animals in the sham control or ischemia-hypothermia groups had cortical lesions (Table 3).

**Table 3:** Number of animals with cortical lesions, white matter lesions, extensive MBP loss and localized diffuse MBP loss

	Cortical lesions	WM lesions	Extensive diffuse MBP loss	Localized diffuse MBP loss
Sham control	0/8	0/8	0/8	0/8
Ischemia-normothermia	7/8*	6/8*	2/8	0/8
Ischemia-hypothermia	0/8	0/8	0/8	5/8*

^*^(*p* < 0.05), Kruskal–Wallis one-way ANOVA

**Fig. 3:**
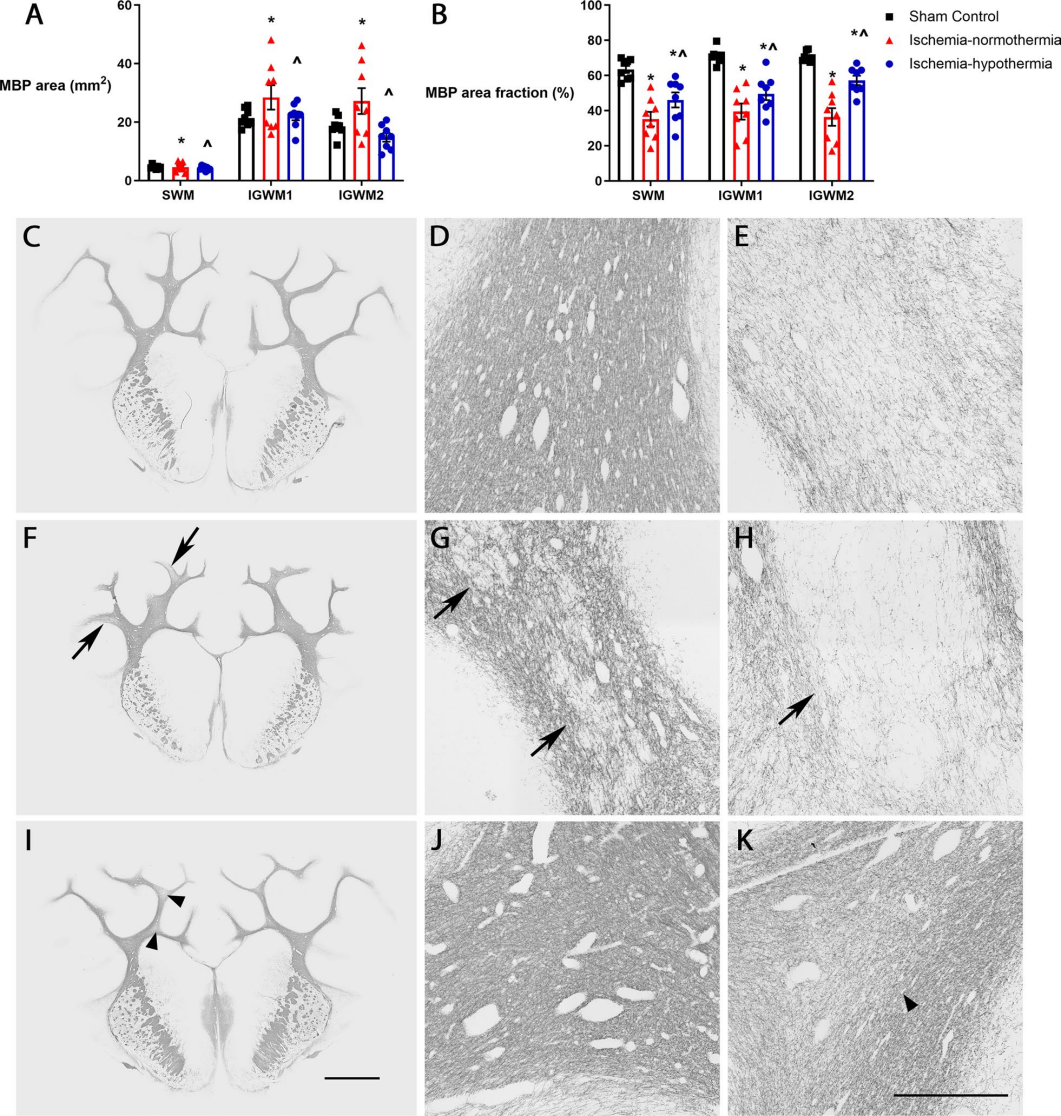
White matter MBP area and area fraction measurements and white matter lesions. **A** Graph showing MBP area measurements and **B** MBP area fraction for the intragyral white matter of the sagittal gyrus (SWM) and the first parasagittal gyrus (IGWM1) and second parasagittal gyrus (IGWM2). Representative MBP whole section images for **C** sham control (*n* = 8), **F** ischemia-normothermia (*n* = 8) and **I** ischemia-hypothermia (*n* = 8). Representative images for **D** intact MBP in sham control, **E** extensive diffuse MBP loss in ischemia-normothermia, **G** small lesions in ischemia-normothermia, **H** large lesions in ischemia-normothermia, **J** intact MBP in ischemia-hypothermia and **K** localized diffuse MBP loss in ischemia-hypothermia. Arrows with tails indicate lesions. Arrow heads without tails indicate areas of localized diffuse MBP loss. *(*p* < 0.05) vs. sham control, ^^^(*p* < 0.05) vs. ischemia-normothermia, repeated measures ANOVA and LSD post hoc test. Scale bar for **I** = 5 mm, scale bar for **K** = 500 µm

There was a significant increase in MBP-labeled white matter area in the ischemia-normothermia group, compared with sham control (*p* = 0.042; Fig. 3A), which was significantly attenuated in the ischemia-hypothermia group (*p* = 0.015). However, the area fraction of MBP labeling was significantly reduced in ischemia-normothermia animals in all areas compared with sham control (Fig. 3B; *p* = 0.001). This was partially attenuated with ischemia-hypothermia (*p* = 0.005) but remained significantly lower than sham control (*p* = 0.001).

MBP labeling was dense and uniform in the intragyral white matter of the sagittal and parasagittal gyri in the sham control group (Fig. 3D). Extensive diffuse myelin loss was evident throughout the intragyral white matter tracts in 2/8 ischemia-normothermia animals (Fig. 3E; Table 3). White matter lesions were only found in the ischemia-normothermia group (6/8 animals, *p* = 0.001). Lesions were predominantly located in the top of the intragyral white matter tracts of the parasagittal gyri. These ranged from multiple small lesions (Fig. 3G), to large lesions (Fig. 3H). Localized diffuse white matter loss was present in 5/8 animals in the ischemia-hypothermia group (Fig. 3K; *p* = 0.001). The remaining 3/8 ischemia-hypothermia animals (Fig. 3J) had similar MBP labeling to sham control on gross appearance (Fig. 3D).

### Microglia

**Fig. 4:**
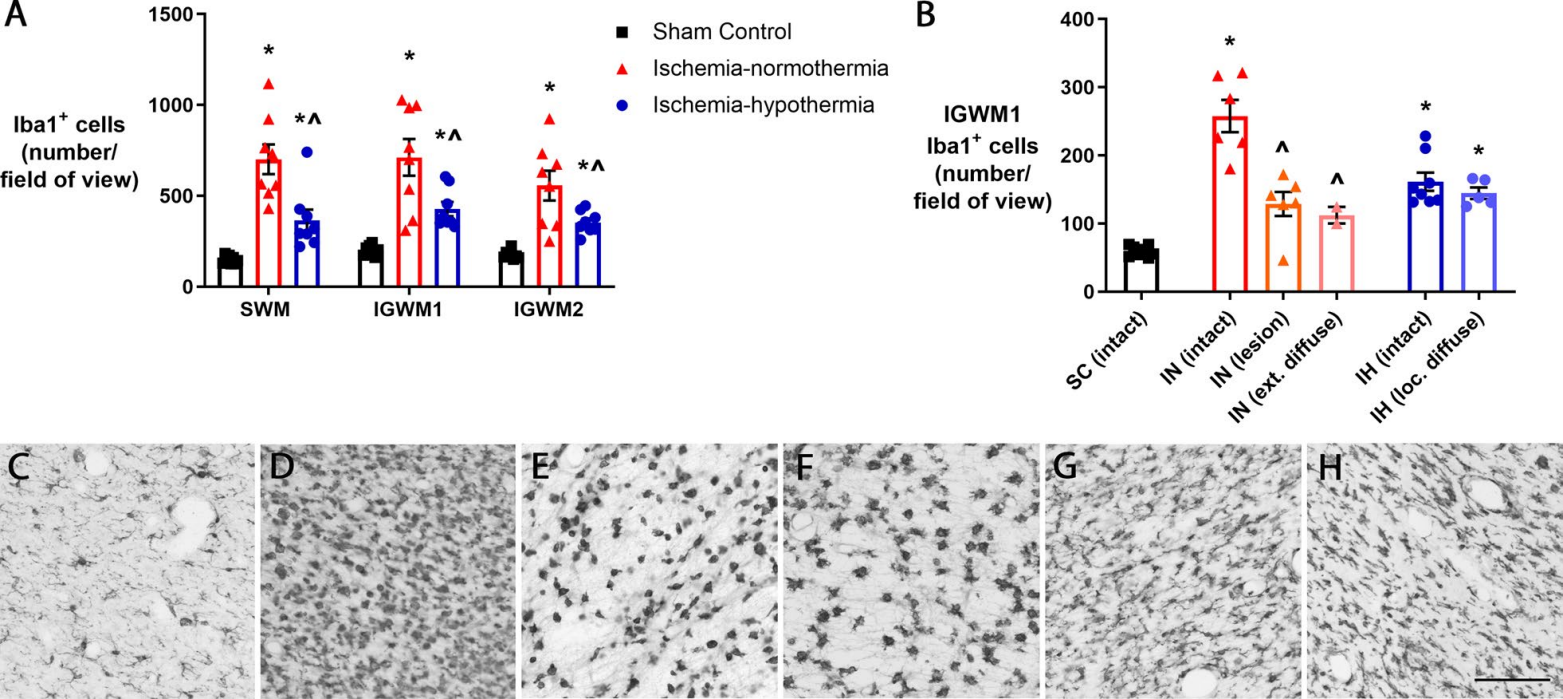
Numbers of microglia in the white matter regions, overall, and stratified by severity of myelin loss. **A** Graph showing number of Iba1-positive cells in the intragyral white matter of the sagittal gyrus (SWM) and the first parasagittal gyrus (IGWM1) and second parasagittal gyrus (IGWM2) in sham control (*n* = 8), ischemia-normothermia (*n* = 8) and ischemia-hypothermia (*n* = 8) groups. *(*p* < 0.05) vs. sham control, ^^^(*p* < 0.05) vs. ischemia-normothermia, repeated measures ANOVA and LSD post hoc test. **B** Graph showing number of Iba1-positive cells in the intact area in sham control (SC), intact area, lesion area and extensive diffuse area in ischemia-normothermia (IN) and intact and localized diffuse area in ischemia-hypothermia (IH). *(*p* < 0.05) vs. sham control intact, ^^^(*p* < 0.05) vs. ischemia-normothermia intact, Kruskal–Wallis one-way ANOVA. Representative images of Iba1 in **C** the intact area in sham control, **D** intact area in ischemia-normothermia, **E** lesion area in ischemia-normothermia and **F** extensive diffuse area in ischemia-normothermia and **G** intact area in ischemia-hypothermia and **H** localized diffuse area in ischemia-hypothermia. Scale bar = 100 µm

There was a significant increase in numbers of microglia in the ischemia-normothermia group, compared with sham control in the intragyral white matter of the sagittal and first and second parasagittal gyri (Fig. 4A; *p* = 0.001). The ischemia-hypothermia group showed intermediate values, which were significantly fewer than ischemia-normothermia (*p* = 0.001) but more than the sham controls (*p* = 0.007). There was differential expression of microglia related to the severity of MBP loss, such that the greatest number of microglia in the white matter were found in the areas of intact MBP in the ischemia-normothermia group (Fig. 4B). In the ischemia-normothermia group, in the white matter lesions (*p* = 0.008) and areas of extensive diffuse MBP loss (*p* = 0.011), there were fewer microglia than in the intact myelin area. In the ischemia-hypothermia group, numbers of microglia were not significantly different between the areas of intact MBP or areas of localized diffuse MBP loss (*p* = 0.737), but still significantly higher than sham control (*p* = 0.001).

**Fig. 5:**
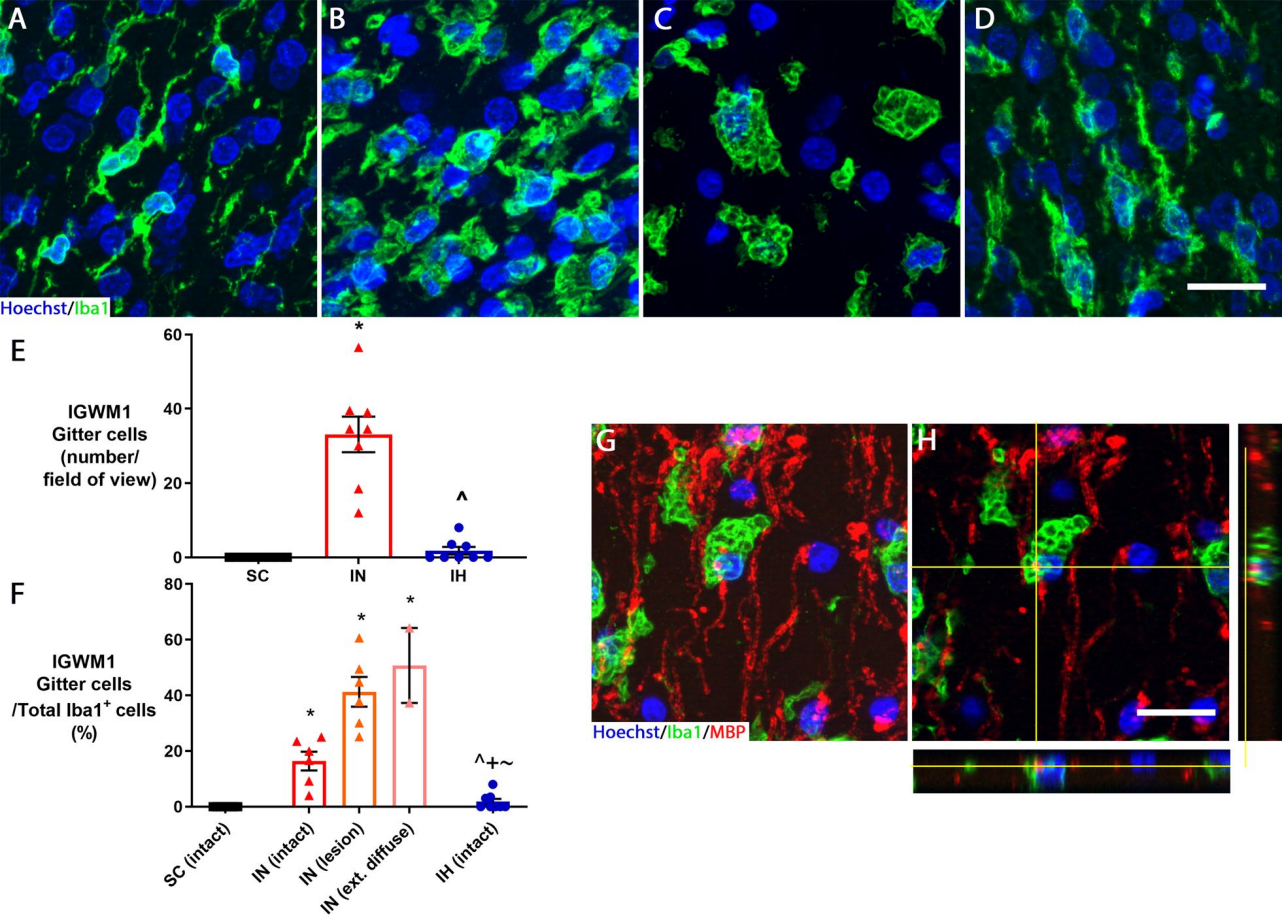
Gitter cells in white matter regions and stratified by severity of myelin loss. Representative images of **A** ramified microglia in sham control, **B** amoeboid microglia in ischemia-normothermia, **C** gitter cells in ischemia-normothermia, **D** ramified microglia in ischemia-hypothermia. **E** Graph showing the number of gitter cells in the sham control (SC) (*n* = 8), ischemia-normothermia (IN) (*n* = 8) and ischemia-hypothermia groups (IH) (*n* = 8) in the intragyral white matter of the first parasagittal gyrus. *(*p* < 0.05) vs. sham control, ^^^(*p* < 0.05) vs. ischemia-normothermia. **F** Graph showing the proportion of gitter cells to the overall number of Iba1-positive cells in the intact area in sham control, lesion area and extensive diffuse area in ischemia-normothermia (IN) and intact area in ischemia-hypothermia (IH). *(*p* < 0.05) vs. sham control intact, ^^^(*p* < 0.05) vs. ischemia-normothermia intact, ^+^(*p* < 0.05) vs. ischemia-normothermia lesion, ^~^(*p* < 0.05) vs. ischemia-normothermia extensive diffuse, Kruskal–Wallis one-way ANOVA for **E** and **F**. **G** Maximum intensity Z-projection of z-stack for gitter cell and MBP labeling. **H** Z-stack slice and associated orthogonal view of gitter cell shown in **G** containing MBP fragments. Scale bar for **D** and **G** = 20 µm

In the sham control group, the microglia had a ramified appearance, with a small cell body with many processes extending from it (Fig. 5A). In the ischemia-normothermia group, qualitatively, many of the microglia appeared to have an amoeboid morphology, with a large cell body and round appearance with retracted processes (Fig. 5B). Further, in the ischemia-normothermia group, there was an abundance of gitter cells (Fig. 5C). In the ischemia-hypothermia group, qualitatively, the microglia showed a more ramified appearance (Fig. 5D).

No gitter cells were seen in the sham control group. The highest number of gitter cells was found in the ischemia-normothermia group, which was significantly higher than sham control (Fig. 5E; *p* = 0.001). Hypothermia reduced the number of gitter cells to sham control levels (*p* = 0.355). The proportion of gitter cells relative to the total number of microglial cells was significantly higher in the intact, lesion and extensive diffuse MBP loss areas compared with the intact areas in the ischemia-normothermia group (Fig. 5F; *p* < 0.008, *p* = 0.001, *p* = 0.002). The intact MBP area in the ischemia-hypothermia group had a significantly lower proportion of gitter cells compared with any area in the ischemia-normothermia group (*p* = 0.050, *p* = 0.001, *p* = 0.010). Z-stacking of sections labeled with Hoechst, Iba1 and MBP show that some gitter cells contained MBP fragments inside the cell (Fig. 5G), which was confirmed in the orthogonal views (Fig. 5H).

**Fig. 6:**
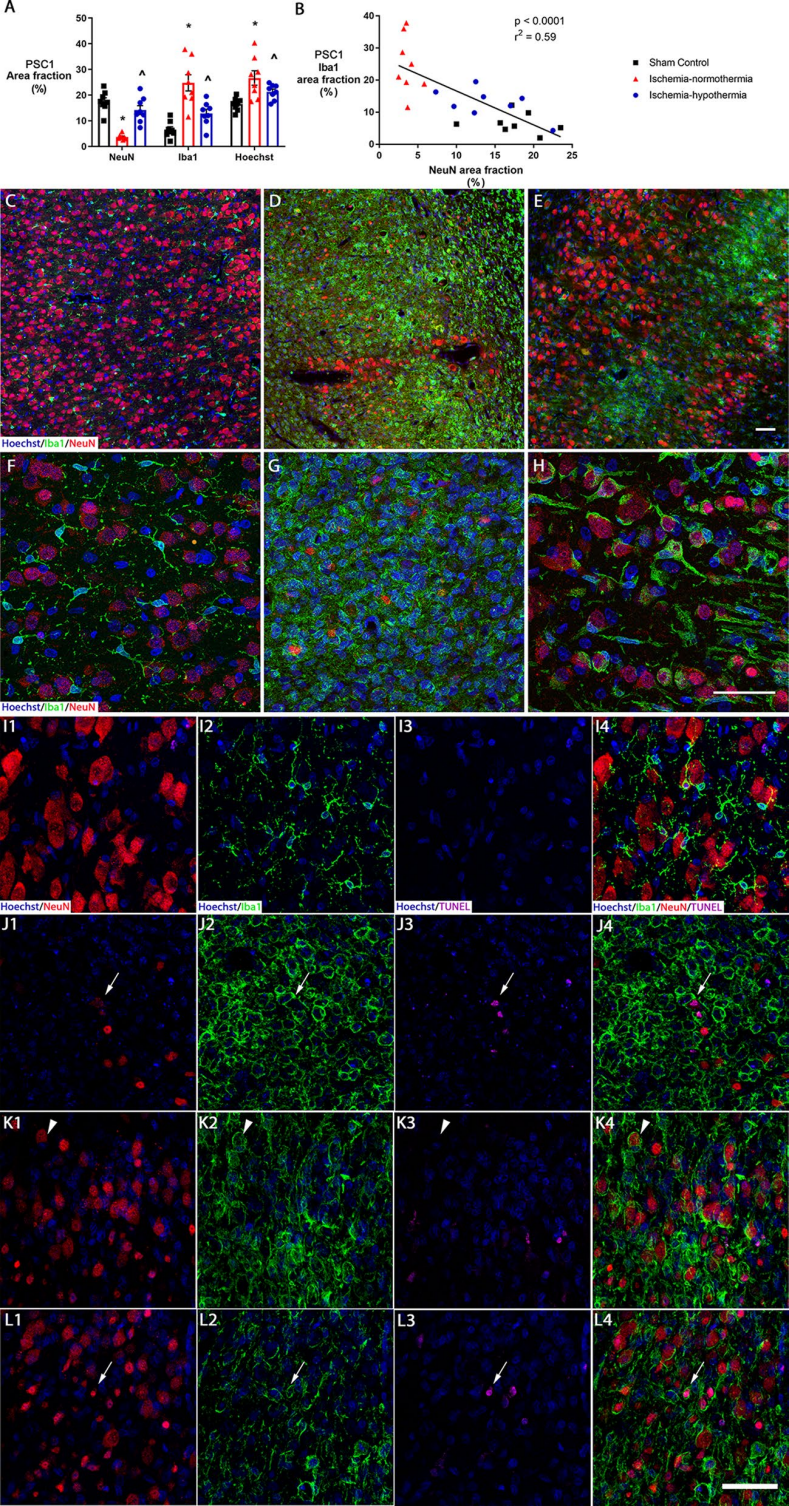
Distribution of cortical microglia and microglial wrapping. **A** Graph showing the area fraction of NeuN, Iba1 and Hoechst in the cortex of the first parasagittal gyrus in sham control (*n* = 8), ischemia-normothermia (*n* = 8) and ischemia-hypothermia (*n* = 8) groups. *(*p* < 0.05) vs. sham control, ^^^(*p* < 0.05) vs. ischemia-normothermia, one-way ANOVA and LSD post hoc test. **B** Graph showing the correlation between NeuN area fraction and Iba1 area fraction in the cortex of the first parasagittal gyrus, linear regression. Representative images of Hoechst, Iba1 and NeuN triple-labeling for **C** sham control with many healthy neurons and few microglia, **D** ischemia-normothermia with few surviving neurons and an abundance of microglia and **E** ischemia-hypothermia with many healthy neurons and some microglia. Representative images of maximum intensity projections of z-stacks for Hoechst, Iba1 and NeuN triple labeling for **F** sham control microglial processes interacting with neurons, **G** ischemia-normothermia showing the abundance of microglia in close proximity to neurons and **H** ischemia-hypothermia showing microglial wrapping. Representative images of maximum intensity projections of z-stacks for Hoechst, Iba1, NeuN and TUNEL quadruple labeling for **I** sham control showing TUNEL negative healthy neurons and ramified microglia, **J** ischemia-normothermia showing TUNEL-positive neurons wrapped by microglia (arrow with tail) and **K** ischemia-hypothermia showing TUNEL negative neurons wrapped by microglia (arrow with no tail) and **L** ischemia-hypothermia showing TUNEL-positive neurons wrapped by microglia (arrow with tail). (1) Hoechst and NeuN, (2) Hoechst and Iba1, (3) Hoechst and TUNEL and (4) all 4 channels merged. Scale bar for H and L4 = 50 µm

In sham control animals, there was a high area fraction of NeuN labeling and low area fraction of Iba1 labeling (Fig. 6A and C). In the ischemia-normothermia animals, there was a reduction in NeuN area fraction and an increase in Iba1 area fraction, compared with sham control in the cortex of the first parasagittal gyrus (Fig. 6D; *p* = 0.001). In the ischemia-hypothermia group, NeuN area fraction (*p* = 0.083) and Iba1 area fraction (*p* = 0.051) were attenuated to sham control levels (Fig. 6E). Decreasing NeuN area fraction was significantly correlated with increasing Iba1 area fraction (Fig. 6B; *p* = 0.0001). Notably, in ischemia-normothermia and ischemia-hypothermia animals, microglia were generally clustered in areas with few neurons.

In sham controls, mainly only microglial processes were in contact with neurons (Fig. 6F). In the ischemia-normothermia animals, there was a high abundance of microglia in close proximity to the neurons (Fig. 6G). In the ischemia-hypothermia group, there was an abundance of microglial wrapping, where the microglial processes or cell body were wrapped around the neuron (Fig. 6H). Confocal images of the co-labeling of neurons, microglia and TUNEL staining showed that in sham control, there was little to no TUNEL staining and no microglial wrapping (Fig. 6I). After ischemia-normothermia, there was some microglial wrapping of neurons and many of these neurons were much smaller and were TUNEL positive (Fig. 6J). Interestingly, the NeuN expression of these TUNEL-positive neurons also appeared to be decreased. In ischemia-hypothermia, most microglial wrapped neurons showed a similar appearance to healthy sham control neurons and were TUNEL negative (Fig. 6K), however, there were some that were TUNEL positive and these neurons were smaller in size (Fig. 6K).

### RNAscope^®^

**Fig. 7:**
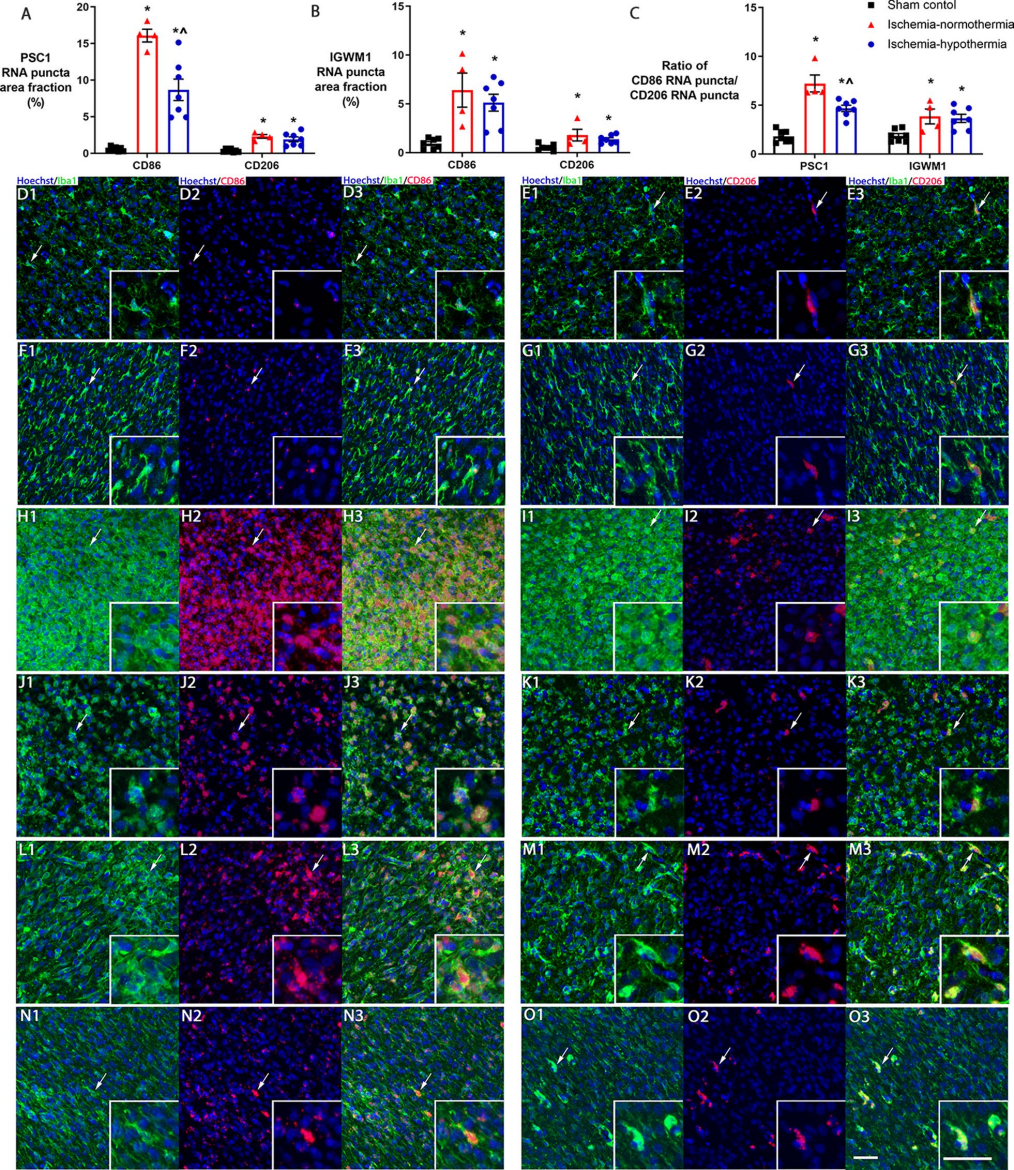
Microglial phenotype marker expression in the cortex and white matter. Graphs showing area fraction of *CD86* and *CD206* RNA puncta in the **A** cortex (PSC) and **B** intragyral white matter of the first parasagittal gyrus (IGWM) in sham control (*n* = 7), ischemia-normothermia (*n* = 4) and ischemia-hypothermia (*n* = 7) groups. **C** Graph showing the ratio of *CD86* puncta to *CD206* puncta in the PSC and IGWM. Representative images of Hoechst, Iba1 and *CD86* for sham control **D** in the PSC and **F** in the IGWM and representative images of Hoechst, Iba1 and *CD206* in **E** in the PSC and **G** in the IGWM showing low expression of both markers. *(*p* < 0.05) vs. sham control, ^^^(*p* < 0.05) vs. ischemia-normothermia, one-way ANOVA and LSD post hoc test. Representative images of Hoechst, Iba1 and *CD86* for ischemia-normothermia **H** in the PSC and **J** in the IGWM and representative images of Hoechst, Iba1 and *CD206* in **I** in the PSC and **K** in the IGWM showing the upregulation of both markers compared with sham control. Representative images of Hoechst, Iba1 and *CD86* for ischemia-hypothermia **L** in the PSC and **N** in the IGWM and representative images of Hoechst, Iba1 and *CD206* in **M** in the PSC and **O** in the IGWM showing the upregulation of both markers compared with sham control but the suppression of *CD86* in the PSC only compared with ischemia-normothermia. Arrows indicate the cell shown in each respective inset. Scale bar for *O* and inset = 50 µm

Area fraction measurements showed that in sham control animals, there was low level expression of both *CD86* and *CD206* in the cortex and intragyral white matter of the parasagittal gyrus (Fig. 7A and B). After ischemia-normothermia there was a significant upregulation of both *CD86* and *CD206*, compared with sham control in both the cortex (*p* = 0.001, *p* = 0.001) and white matter of the first parasagittal gyrus (*p* = 0.001, *p* = 0.005). Hypothermia partially attenuated *CD86* expression in the cortex only (*p* = 0.001), and had no effect on the expression of *CD206* compared with ischemia-normothermia (*p* = 0.330).

In sham control animals, there was slightly higher *CD86* expression compared with *CD206* (Fig. 7C). Ischemia was associated with a significant increase in the ratio of *CD86* to *CD206* expression in both areas compared with sham control (*p* = 0.001). There was a significant increase in the ratio of *CD86* to *CD206* expression in the ischemia-hypothermia group in both areas compared with sham control (*p* = 0.001). However, in the cortex of the parasagittal gyrus, the ischemia-hypothermia group showed a reduced ratio of *CD86* to *CD206* compared with ischemia-normothermia (*p* = 0.001).

RNAscope^®^ combined with immunohistochemistry showed that both *CD86* and *CD206* were expressed by Iba1-positive microglia in all groups (Fig. 7D–O). Qualitatively, in sham controls, *CD86* was lowly expressed (with few puncta per cell) across more microglia, whereas *CD206* was highly expressed (many puncta per cell) across fewer microglia. In the ischemia-normothermia group, the expression of *CD86* was higher within each cell and was present across many cells, whereas the expression of *CD206* within each cell showed a mixture of high and low expression and there were more microglia that expressed *CD206*, compared with sham control. The expression pattern of *CD86* in ischemia-hypothermia animals was a mixture of high and low expression within each cell but in many fewer cells and the expression pattern for *CD206* was similar to ischemia-normothermia.

### Astrocytes

**Fig. 8:**
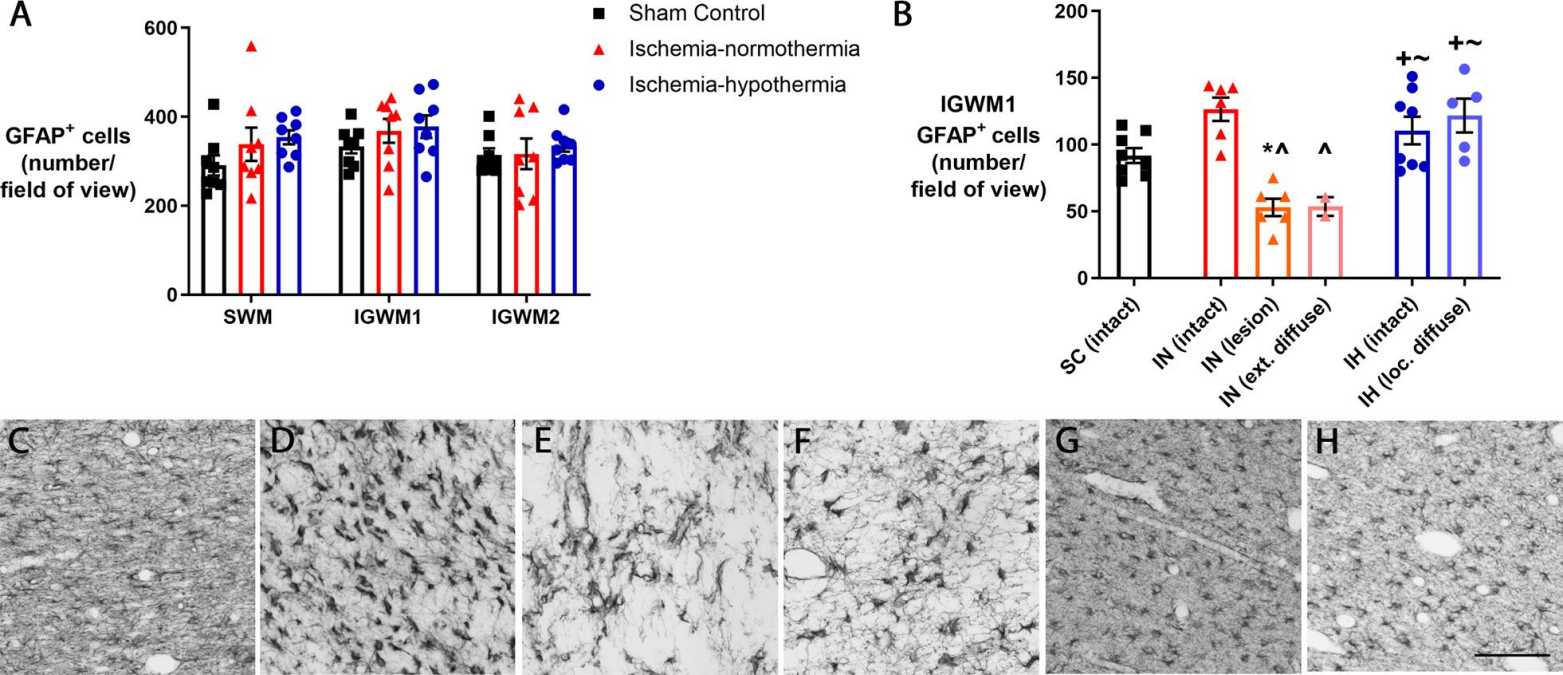
Astrocyte number in the white matter and stratified by severity of myelin loss. **A** Graph showing number of GFAP-positive cells in the in the intragyral white matter of the sagittal (SWM) first parasagittal (IGWM1) and second parasagittal gyri (IGWM2) in sham control (*n* = 8), ischemia-normothermia (*n* = 8) and ischemia-hypothermia (*n* = 8) groups, repeated measures ANOVA. **B** Graph showing number of GFAP-positive cells in the intact area in sham control (SC), intact area, lesion area and extensive diffuse area in ischemia-normothermia (IN) and intact and localized diffuse area in ischemia-hypothermia (IH), Kruskal–Wallis one-way ANOVA. *(*p* < 0.05) vs. sham control intact, ^^^(*p* < 0.05) vs. ischemia-normothermia intact, ^+^(*p* < 0.05) vs. ischemia-normothermia lesion, ^~^(*p* < 0.05) vs. ischemia-normothermia extensive diffuse. Representative images of GFAP in **C** the intact area in sham control, **D** intact area in ischemia-normothermia, **E** lesion area in ischemia-normothermia and **F** extensive diffuse area in ischemia-normothermia and **G** intact area in ischemia-hypothermia and **H** localized diffuse area in ischemia-hypothermia. Scale bar = 100 µm

The number of GFAP-positive astrocytes in the intragyral white matter of the sagittal and parasagittal gyri were not significantly different between groups (Fig. 8A; *p* = 0.356). In the ischemia-normothermia lesion and extensive diffuse MBP loss areas, the number of astrocytes were significantly lower than ischemia-normothermia intact areas (Fig. 8B; *p* = 0.001, *p* = 0.007). In the ischemia-hypothermia intact and localized diffuse MBP loss areas, astrocyte number was significantly higher than in the lesion (*p* = 0.002, *p* = 0.001) and extensive diffuse areas (*p* = 0.036, *p* = 0.015) in the ischemia-normothermia group, but was not significantly different to the intact areas of the sham control (*p* = 0.317, *p* = 0.122) or ischemia-normothermia group (*p* = 0.306, *p* = 0.776).

In the sham control group, astrocytes had small cell bodies and long processes (Fig. 8C) but ischemia-normothermia was associated with the hypertrophy of the astrocytic cell bodies and the retraction of processes (Fig. 8D–F). In the ischemia-hypothermia group, the astrocytes have more similar appearance to the sham control group (Fig. 8G, H).

**Fig. 9:**
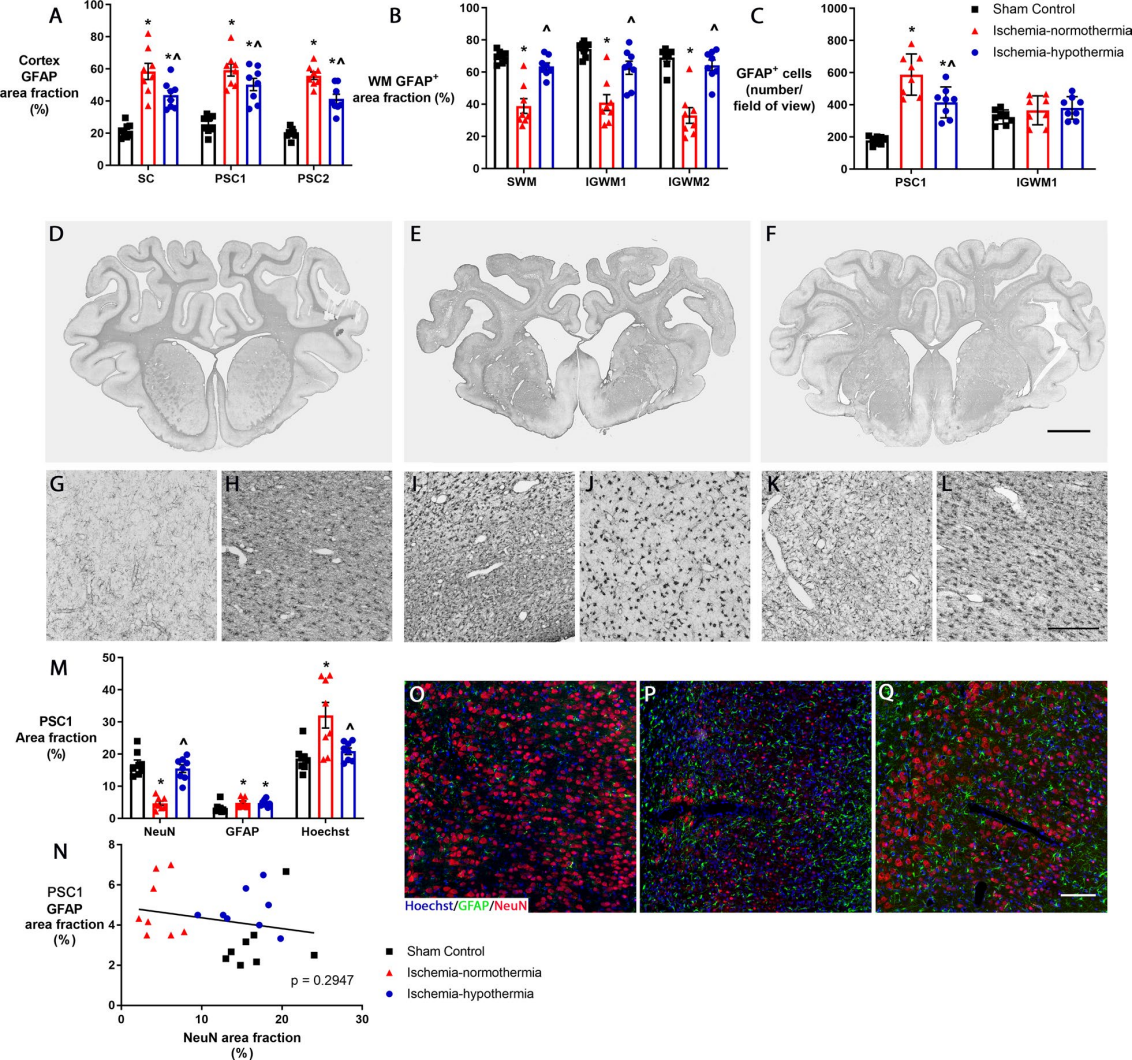
Astrocyte area fraction in the cortex and white matter. **A** Graph showing GFAP area fraction for the cortex of the sagittal (SC), first parasagittal (PSC1) and second parasagittal cortex gyri (PSC2) in sham control (*n* = 8), ischemia-normothermia (*n* = 8) and ischemia-hypothermia (*n* = 8) groups. **B** Graph showing GFAP area fraction in the intragyral white matter of the sagittal (SWM), first parasagittal (IGWM1) and second parasagittal gyri (IGWM2). **C** Graph showing number of GFAP-positive cells in the cortex and intragyral white matter of the first parasagittal gyrus. *(*p* < 0.05) vs. sham control, ^^^(*p* < 0.05) vs. ischemia-normothermia. Repeated measures ANOVA and LSD post hoc test for **A** and **B**, one-way ANOVA and LSD post hoc test for **C**. Representative GFAP whole section images for **D** sham control with higher GFAP expression in the white matter, **E** ischemia-normothermia with increased GFAP expression in the cortex and reduced in the white matter and **F** ischemia-hypothermia with normalization of GFAP expression distribution. Scale bar for **F** = 5 mm. Representative GFAP images for sham control in the **G** cortex and **H** white matter, ischemia-normothermia in the **I** cortex and **J** white matter and ischemia-hypothermia in the **K** cortex and **L** white matter. Scale bar for **L** = 200 µm. **M** Graph showing area fraction for NeuN, GFAP and Hoechst, one-way ANOVA and LSD post hoc test. **N** Graph showing no significant correlation between NeuN area fraction and GFAP area fraction, by linear regression. Representative images of Hoechst, GFAP and NeuN triple-labeling for **O** sham control, **P** ischemia-normothermia and **Q** ischemia-hypothermia. Scale bar for **Q** = 100 µm

The area fraction of GFAP in the cortex of the sagittal and the first and second parasagittal gyri was greater in the ischemia-normothermia group than sham controls (Fig. 9A; *p* = 0.001). This increase in GFAP area fraction was partially attenuated to sham control levels in the ischemia-hypothermia group (*p* = 0.001). In the intragyral white matter of the sagittal and the first and second parasagittal gyri, GFAP area fraction was less in the ischemia-normothermia group, than in sham controls (Fig. 9B; *p* = 0.001). GFAP area fraction was restored in the ischemia-hypothermia group to sham control levels (*p* = 0.128).

Numbers of astrocytes in the cortex of the first parasagittal gyrus were lower than in the intragyral white matter in the sham control group (Fig. 9C). Morphologically, these astrocytes in the cortex had a small cell body and many processes (Fig. 9G). In the ischemia-normothermia group, astrocyte number was greater in the cortex of the first parasagittal gyrus compared with the sham control group (*p* = 0.001); morphologically the cells appeared to be hypertrophied, with retracted processes (Fig. 9C and I). In the ischemia-hypothermia group, there was a partial attenuation of numbers of astrocytes compared with ischemia-normothermia (*p* < 0.001) and the astrocyte cell bodies appeared hypertrophied and had thicker processes than in sham controls (Fig. 9K).

There was no significant correlation between GFAP area fraction and NeuN area fraction in the cortex of the first parasagittal gyrus (Fig. 9N; *p* = 0.295).

### Correlations of histological changes with EEG power

**Fig. 10:**
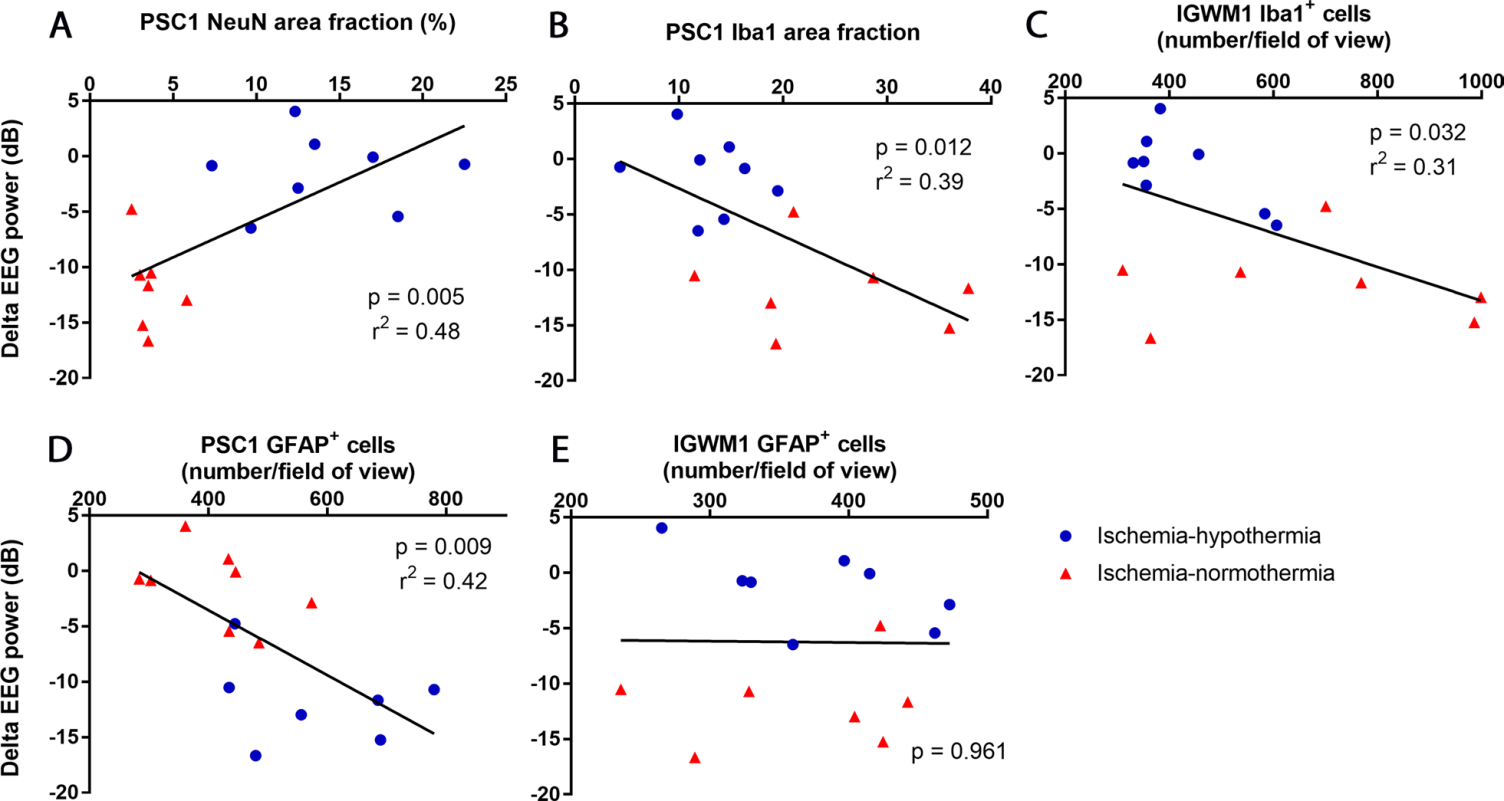
Correlation of neurons, microglia and astrocytes with final EEG power at day 7. Graphs showing correlations of ischemia-normothermia (*n* = 8) and ischemia-hypothermia (*n* = 8) groups between final delta EEG power and **A** NeuN area fraction in the cortex of the first parasagittal gyrus (PSC1), **B** Iba1 area fraction in the cortex of the first parasagittal gyrus, **C** Iba1 cell number in the intragyral white matter of the first parasagittal gyrus (IGWM1), **D** GFAP cell number in the cortex of the first parasagittal gyrus and **E** GFAP cell number in the intragyral white matter of the first parasagittal gyrus. Linear regression was used for all correlations

Neuronal area fraction in the cortex of the first parasagittal gyrus was significantly correlated with final EEG power at day 7 (Fig. 10; *p* = 0.005). Greater microglial area fraction in the cortex (*p* = 0.012) and increased numbers of microglia (*p* = 0.032) in the intragyral white matter of the first parasagittal gyrus were inversely correlated with final EEG power at day 7. Numbers of astrocytes in the cortex (*p* = 0.009) but not in the intragyral white matter of the first parasagittal gyrus (*p* = 0.961), were inversely correlated with final EEG power at day 7.

## Discussion

This study demonstrates that after global ischemia in near-term fetal sheep, a clinical protocol of delayed but prolonged therapeutic hypothermia prevented formation of cortical and white matter lesions but was associated with only partial attenuation of microgliosis and of the ratio of M1:M2 marker expression. Interestingly, numbers of cortical and white matter microglia and cortical astrocytes were closely and inversely proportional to EEG power at day 7, raising the possibility that neuroinflammation may contribute to impaired functional recovery. These data support the concept that microglia activation after therapeutic hypothermia may be an important therapeutic target that should tested in future studies.

### Hypothermia reduced cortical area loss, white matter swelling and prevented lesions

Cerebral ischemia was consistently associated with formation of cortical and white matter lesions after 7 days of recovery. Similar lesions have been reported in human term infants after HIE using MRI [28, 29], and in the cortex of near-term fetal sheep after cerebral ischemia [30]. We now report that white matter lesions also developed after global cerebral ischemia. Interestingly, in preterm fetal sheep exposed to severe asphyxia, white matter lesions developed very slowly and only appeared after 2 weeks of recovery [31]. Speculatively, the more rapid appearance of lesions in the present study may be related to either greater brain maturation, or greater severity of injury. Overall cortical area was reduced by ischemia-normothermia, with severe neuronal loss and reduced brain weight. By contrast, there was significant macroscopic swelling of the white matter tracts after ischemia, similar to our previous report [32], as shown by a greater area of MBP labeling, despite a lower area fraction of MBP compared with sham controls.

Strikingly, in the present study, therapeutic hypothermia started 3 h after ischemia completely prevented the formation of cortical and white matter lesions and normalized cortical and white matter area to sham control levels. In the present study, localized diffuse myelin loss was still present after therapeutic hypothermia in some areas. It is unclear whether these subtle changes would be detected with routine MRI. In human infants who received therapeutic hypothermia for moderate-to-severe HIE, MRI studies have consistently shown a significant but partial reduction in the number of white and gray matter lesions [1, 2, 33]. This difference may in part be related to greater delay after the start of HI before starting therapeutic hypothermia in a clinical setting. For example, in a large cohort of infants with moderate-to-severe HIE, cooling started before 3 h of birth was associated with better motor outcomes than when cooling was started after 3 h [34].

### Differential distribution of numbers of microglia relative to severity of myelin loss in the white matter

Microglia are markedly upregulated after cerebral ischemia in near-term fetal sheep and at least partially suppressed after hypothermia [8, 9]. In the present study, we found that the relationship with injury was not homogenous within animals, such that clusters of microglia were prevalent around areas of high neuronal loss, but overt lesions contained fewer microglia. Ischemia-normothermia was associated with increased numbers of gitter cells. Gitter cells are microglia that have phagocytosed debris or fat particles and have a lattice-like-appearance due to the cytoplasm containing many vacuoles [35]. Other studies have shown that phagocytically active microglia/macrophages containing myelin debris or lysosomal lipids are seen in active inflammatory brain lesions in patients with multiple sclerosis and in rats with experimental autoimmune encephalomyelitis [36, 37]. The importance of myelin debris removal by gitter cells after ischemia is unclear, but they may promote remyelination [38].

### Proliferation of microglia in the cortex and microglial wrapping

In the present study, in sham controls, microglial processes interacted closely with neurons, consistent with a role in monitoring neurons under physiological conditions [39]. After ischemia, microglia in the cortex wrapped around the neuronal cell bodies. This is likely mediated by microglia detecting signaling molecules such as adenosine triphosphate (ATP), which can be released by neurons after ischemia [40]. Wrapping has been reported after a variety of neural injuries including oxygen glucose deprivation in hippocampal slice cultures [41] and cerebral inflammation induced by stereotaxic injection of killed bacteria in adult rats [42].

Most studies suggest that microglial wrapping may be an endogenous neuroprotective mechanism to restore neuronal function and to isolate and phagocytose the dying/dead neuron and so protect surrounding neurons from exposure to the cell contents or debris released from the dying/dead neuron [40, 43]. When immortalized murine microglial cells (BV-2) were applied to organotypic hippocampal slice cultures after oxygen glucose deprivation, the microglia migrated to wrap around the neurons and this resulted in reduced neuronal damage, assessed at 24 h after the insult [41]. One of the mechanisms of protection may be microglial-mediated stripping of presynaptic terminals at the neuronal cell body, as shown in models of cerebral inflammation [42, 44], and associated with increased expression of anti-apoptotic and neurotrophic molecules and reduced cortical neuron apoptosis [44].

The timing of microglial wrapping after ischemia is likely to determine its impact. For example, BV2 microglia were only neuroprotective when applied within 4 h after oxygen glucose deprivation in hippocampal slice cultures but not at 6 h [41]. However, the time course and effect of microglial wrapping persisting to day 7 in the present study is not known. We found that some of the wrapped neurons were TUNEL negative. Thus, it is possible that microglial wrapping may have helped to promote neuronal survival in some cases. Functionally, microglia wrapping can displace synapses [42, 44], raising the possibility that it may reduce neuronal connectivity, and so may impair EEG recovery.

### Hypothermia partially suppressed “pro-inflammatory” microglial phenotype in the cortex

In this large animal, translational model of perinatal HI, there was a profound upregulation of both *CD86* and *CD206* mRNA in the cortex and white matter of the parasagittal gyrus after 7 days recovery. By contrast, in P9 rats, upregulation of *CD86*-positive cells was seen from 24 h to 7 days after common carotid artery ligation and hypoxia, but upregulation of *CD206*-positive cells was present at 24 and 72 h but not 7 days [16]. These differences in the time course of M1/M2 polarization between studies are likely related to differences in species and model; for example, that the rodent HI model leads to a severe stroke-like pattern of damage. Interestingly, there is growing evidence from rodent studies that inflammation and microglial responses to HI can be modulated in a sex-dependent manner [45]. However, the present study was not powered to assess sex differences.

In the present study, hypothermia was associated with partial downregulation of *CD86* mRNA and the ratio of *CD86* to *CD206* expression in the cortex of the parasagittal gyrus, consistent with an anti-inflammatory role for hypothermia [46]. By contrast, in the white matter, there was no significant effect of ischemia-hypothermia on the ratio of *CD86* to *CD206* compared with ischemia-normothermia. Potentially, this may be related to partial protection of oligodendrocytes with hypothermia starting 3 h after the end of ischemia [8]. We speculate that maximal hypothermia-mediated shift to M2 polarization in white matter may only be achieved with earlier initiation of hypothermia. This hypothesis is consistent with evidence in P9 mice that after common carotid ligation and hypoxia, hypothermia started immediately after the insult was associated with downregulated CD86 expression at day 1 [17].

### Hypothermia partially normalized astrocyte number and morphology

There was a differential astrocytic response in the cortex compared with the white matter in the present study. In the white matter, after ischemia there was no change in numbers of astrocytes but there was hypertrophy of the cell body and a loss of GFAP-positive processes shown by reduced area fraction, consistent with reactive astrogliosis [47]. These morphological changes were similar to those at 3 days after hypoxia in the neonatal piglet [48]. Importantly, Sullivan et al., showed that changes in GFAP labeling was not simply a change in GFAP expression by astrocytes, but was also correlated with altered morphology shown with Golgi–Kopsch staining [48].

In the present study, cortical astrocytes were also hypertrophied with retracted processes, but unlike in the white matter, numbers of astrocytes were increased. Hypothermia was associated with at least a partial attenuation of reactive astrogliosis in white and gray matter.

Similar to numbers of microglia, there were reduced numbers of astrocytes in the white matter lesions and in areas showing extensive diffuse MBP loss, compared with intact regions. This may have reflected necrosis of tissue within parts of the lesions, with loss of all cell types. Microglia and astrocytes were mostly present in small “threads” of remaining tissue inside the lesion and the edges of intact tissue.

### Inflammation and EEG recovery

Both cortical neuronal survival and the extent of inflammation after ischemia were closely correlated with EEG recovery at day 7. Microglial area fraction was inversely proportional to both neuronal area fraction as well as EEG power in the cortex, suggesting that the deficit in EEG power after ischemia-hypothermia in the present study may be related to a combination of mild residual neuronal loss and persistent microgliosis. Further, increased microgliosis in the white matter was also associated with worse EEG recovery, potentially related to the severity of axonal injury [10]. Of interest, numbers of cortical astrocytes were independently correlated with EEG recovery, but not neuronal area fraction, potentially suggesting a neuromodulatory effect of the astrocyte reaction.

### Limitations

The reader should consider some limitations of the present study. Lesions were defined visually on serial brain sections labeled with either MBP, Iba1 or GFAP. However, the total volume of the lesions could not be quantified without labeling a substantial number of sections to encompass the lesion. This was not possible, as we had limited tissue available.

Further, only two microglial phenotype markers were examined. Microglia are highly pleomorphic, and so it is plausible that other phenotypes beyond the classic M1/M2 dichotomy may have been present [24]. Additionally, RNA detection by RNAscope^®^ is affected by the age of the formalin-fixed, paraffin-embedded tissue block [49], and so only a subset of animals in this study were able to be reliably assessed by RNAscope^®^. Therefore, we were unable to correlate the expression of M1 and M2 markers with the severity of MBP loss.

Lastly, microglia and astrocytes were assessed after 7 days recovery. Neuroinflammation is a dynamic process and so further time points will need to be assessed in the future to characterize the ultimate recovery of gray and white matter [31].

## Conclusions

Treatment with therapeutic hypothermia started at 3 h after global cerebral ischemia prevented cortical and white matter lesions in near-term fetal sheep after 7 days recovery, but only partially suppressed microglial wrapping and M1 marker expression. These data suggest the hypothesis that persistent upregulation of injurious microglial activity may contribute to partial neuroprotection after hypothermia, and that immunomodulation after rewarming may be an important therapeutic target to be tested in future preclinical studies.

## Data Availability

The datasets used and/or analyzed during the current study are available from the corresponding author on reasonable request.

## Abbreviations

HIE: Hypoxic-ischemic encephalopathy
HI: Hypoxia-ischemia
P: Postnatal day
MRI: Magnetic resonance imaging
ATP: Adenosine triphosphate
MCAO: Middle cerebral artery occlusion
IL: Interleukin
TGF: Transforming growth factor
CD: Cluster of differentiation
NOS: Nitric oxide synthase
IGF: Insulin-like growth factor
SR-A: Macrophage scavenger receptor
